# In vitro optimization and comparison of CT angiography versus radial cardiovascular magnetic resonance for the quantification of cross-sectional areas and coronary endothelial function

**DOI:** 10.1186/s12968-019-0521-z

**Published:** 2019-02-07

**Authors:** Jérôme Yerly, Fabio Becce, Ruud B. van Heeswijk, Francis R. Verdun, Danilo Gubian, Reto Meuli, Matthias Stuber

**Affiliations:** 1 Department of Diagnostic and Interventional Radiology, Lausanne University Hospital (CHUV and UNIL), Rue du Bugnon 46, Lausanne, 1011 VD Switzerland; 2 Center for Biomedical Imaging (CIBM), Lausanne, Switzerland; 3 Institute of Radiation Physics, Lausanne University Hospital (CHUV and UNIL), Lausanne, Switzerland; 4 Direction des Constructions, Ingénierie, Technique et Sécurité (CIT-S), Lausanne University Hospital (CHUV and UNIL), Lausanne, Switzerland

**Keywords:** Accuracy, CT angiography, Coronary artery, Cross-sectional area, Endothelial function, Limit of detection, Precision, Radial CMR, Vasodilation, Vasomotor response

## Abstract

**Background:**

Our objectives were first to determine the optimal coronary computed tomography angiography (CTA) protocol for the quantification and detection of simulated coronary artery cross-sectional area (CSA) differences in vitro, and secondly to quantitatively compare the performance of the optimized CTA protocol with a previously validated radial coronary cardiovascular magnetic resonance (CMR) technique.

**Methods:**

256-multidetector CTA and radial coronary CMR were used to obtain images of a custom in vitro resolution phantom simulating a range of physiological responses of coronary arteries to stress. CSAs were automatically quantified and compared with known nominal values to determine the accuracy, precision, signal-to-noise ratio (SNR), and circularity of CSA measurements, as well as the limit of detection (LOD) of CSA differences. Various iodine concentrations, radiation dose levels, tube potentials, and iterative image reconstruction algorithms (ASiR-V) were investigated to determine the optimal CTA protocol. The performance of the optimized CTA protocol was then compared with a radial coronary CMR method previously developed for endothelial function assessment under both static and moving conditions.

**Results:**

The iodine concentration, dose level, tube potential, and reconstruction algorithm all had significant effects (all *p* <  0.001) on the accuracy, precision, LOD, SNR, and circularity of CSA measurements with CTA. The best precision, LOD, SNR, and circularity with CTA were achieved with 6% iodine, 20 mGy, 100 kVp, and 90% ASiR-V. Compared with the optimized CTA protocol under static conditions, radial coronary CMR was less accurate (− 0.91 ± 0.13 mm^2^ vs. -0.35 ± 0.04 mm^2^, *p* <  0.001), but more precise (0.08 ± 0.02 mm^2^ vs. 0.21 ± 0.02 mm^2^, *p* <  0.001), and enabled the detection of significantly smaller CSA differences (0.16 ± 0.06 mm^2^ vs. 0.52 ± 0.04 mm^2^; *p* <  0.001; corresponding to CSA percentage differences of 2.3 ± 0.8% vs. 7.4 ± 0.6% for a 3-mm baseline diameter). The same results held true under moving conditions as CSA measurements with CMR were less affected by motion.

**Conclusions:**

Radial coronary CMR was more precise and outperformed CTA for the specific task of detecting small CSA differences in vitro, and was able to reliably identify CSA changes an order of magnitude smaller than those reported for healthy physiological vasomotor responses of proximal coronary arteries. However, CTA yielded more accurate CSA measurements, which may prove useful in other clinical scenarios, such as coronary artery stenosis assessment.

## Background

Measurements of coronary endothelial function (CEF) offer a window into the fundamental pathophysiology of coronary artery disease progression [1–4]. Although the endothelium serves multiple functions, for practical reasons, researchers have thus far investigated the regulation of vascular tone in response to endothelium-dependent stressors as a marker of endothelial health. Using both invasive [2, 5] and non-invasive [6, 7] techniques, healthy coronary arteries have been shown to dilate by approximately 10–25% in response to various endothelium-dependent stressors, whereas reduced dilation and even paradoxical vasoconstriction were observed in impaired coronary arteries [2]. Of note, only relative cross sectional area (CSA) changes need to be measured for assessing CEF and the magnitude of the CSA percentage difference for the accurate differentiation between healthy and diseased coronary artery segments remains uncertain. However, because of their invasive nature, imaging techniques necessitating catheter procedures to measure the CSA of coronary arteries, such as x-ray coronary angiography [2] and intravascular ultrasound [5], are restricted to patients with advanced disease and are not ethically justifiable for repeated use in low-risk subjects. As alternative imaging modalities for the non-invasive measurement of the vasomotor response of coronary arteries, researchers have used cardiovascular magnetic resonance (CMR) [6, 8–11] and computed tomography (CT) [12].

The use of cardiovascular magnetic resonance (CMR) with isometric handgrip exercise [6, 8–11] as an endothelium-dependent stressor to quantify the vasomotor response of coronary arteries was previously shown to yield repeatable results [10]. In addition, radial coronary CMR has recently been shown in vitro to be capable of reliably detecting CSA differences of coronary arteries in the order of 3–4% for images with sufficiently high signal-to-noise ratio (SNR) [13]. Radial coronary CMR seems therefore adequate for measuring CSA differences on the order of magnitude of vasomotor responses observed in healthy adult subjects [11].

While the feasibility of measuring the vasomotor response with CT using the cold pressor test as endothelium-dependent stressor has been demonstrated [12], the performance of CT angiography (CTA) to measure small CSA changes of coronary arteries has yet to be quantitatively determined and compared with CMR. Because CTA offers higher overall temporal and 3D spatial resolution, superior detection of coronary artery stenosis [14, 15], and therefore better overall diagnostic performance than CMR, we hypothesized that CTA would also enable more precise and accurate measurements of coronary artery CSA.

Therefore, the objectives of our study were twofold: first, we aimed to determine the optimal set of CTA data acquisition and image reconstruction parameters for the quantification and detection of simulated coronary artery CSA differences in vitro; and secondly, we compared the performance of the optimized coronary CTA protocol with a previously validated radial coronary CMR technique.

## Methods

### Phantom and experiment design

**Fig. 1:**
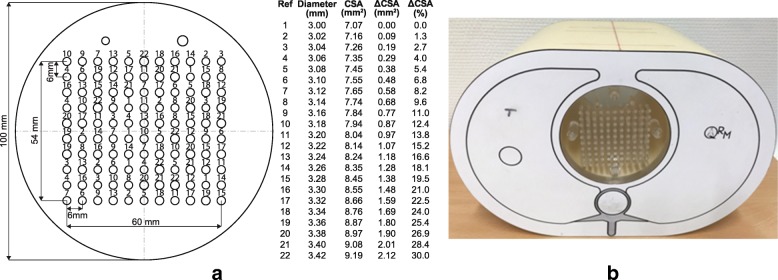
**a** Drilling layout of the coronary artery-mimicking phantom manufactured in a 10-cm-diameter 2-cm-thick polymethyl methacrylate (PMMA) block. The 22 drilled diameters ranged from 3.00–0/+ 0.004 mm to 3.42–0/+ 0.004 mm, in steps of 0.02 mm, and were repeated at 5 different grid locations in the phantom, adding up to a total of 110 holes. **b** For computed tomography angiography (CTA) scans, the PMMA block was inserted into a commercially available anthropomorphic thorax computed tomography (CT) phantom. CSA indicates cross-sectional area; ΔCSA, cross-sectional area difference with 3-mm nominal diameter

A custom 10-cm-diameter 2-cm-thick polymethyl methacrylate (PMMA) resolution phantom was designed to simulate different vessel diameters consistent with the range of vasomotor responses observed in healthy coronary arteries. The phantom was similar to one previously tested [13], yet its shape (round vs. square) and material (PMMA vs. polyoxymethylene copolymer) were adapted to be compatible with anthropomorphic phantom and x-ray attenuation properties. The range of vessel CSAs were simulated by drilling holes with *N*_*d*_ = 22 different diameters, *d* ranging from 3.00–0/+ 0.004 mm to 3.42–0/+ 0.004 mm, in steps of 0.02 mm (Fig. 1a). The extremely high accuracy required for drilling holes also influenced the choice of PMMA for the resolution phantom. Five holes were drilled per diameter at random locations on a grid in the resolution phantom using high-precision reamers (Magafor, Fontenay-sous-Bois, France), for a total of 110 holes.

For CTA, the PMMA resolution phantom was inserted into a commercially available anthropomorphic thorax phantom (QRM, Moehrendorf, Germany, Fig. 1b) and immersed in water blended with iodinated contrast medium (Accupaque 350 mg I/mL, General Electric Healthcare, Little Chalfont, United Kingdom) to simulate various x-ray attenuation differences (in Hounsfield unit, HU) encountered in vivo between coronary arteries and pericardial fat (∆HU range, approximately 400–500 HU at 120 kVp) [16, 17]. Accordingly, a total of four different iodine concentrations were investigated: 3, 4, 5, and 6% (corresponding to 10.5, 14.0, 17.5, and 21.0 mg I/mL) yielding ∆HU of approximately 170, 260, 330, and 390 HU at 120 kVp and 250, 370, 460, and 560 HU at 100 kVp, respectively. This range of x-ray attenuation differences is further consistent with those simulated in a previous in vitro study [18].

For CMR, the coronary artery-mimicking resolution phantom was placed in a container filled with tap water. This water was then doped with 0.5 mmol Gd/mL gadolinium-based contrast agent (Dotarem, Guerbet, Roissy, France) to simulate various in-flow effects of fresh and unsaturated blood entering the slice as in 2D cine imaging. Previous phantom studies have shown that the signal intensity of time-of-flight images no longer depended on flow rate when the blood gadolinium concentration reached approximately 1.0 mmol/L [19]. Therefore, a total of eight different gadolinium concentrations were investigated: 0.00, 0.17, 0.33, 0.50, 0.67, 1.00, 1.33, and 1.66 mmol/L.

### Moving phantom setup

To investigate the effect of motion on CSA measurements on both CMR and CTA, a unidirectional sinusoidal cardiac rhythm was simulated by placing the resolution phantom on a custom-designed moving structure similar to that reported in [20]. The setup was programmed to yield a frequency of 60 beats per minute and a maximum displacement of 3 cm. The setup also provided a triggering signal to synchronize data acquisition with phantom motion. For CTA, only the 6% (21.0 mg I/mL) iodine concentration was used to best simulate x-ray attenuation differences encountered in vivo. For CMR, the gadolinium concentration was adjusted to achieve a SNR of approximately 50, which is similar to that found in vivo [13, 21].

### CTA protocol

CTA scans were performed on a 256-detector row CT system (Revolution CT, General Electric Healthcare, Waukesha, Wisconson, USA) using the axial mode. Parameters for data acquisition and image reconstruction are detailed in Table 1. For data acquisition, three radiation dose levels (volume CT dose indexes, CTDI_vol_, of 5, 10, and 20 mGy) and two tube potentials (100 and 120 kVp) were investigated. Of note, uncommonly slow (for coronary CTA) gantry revolution times (0.5 and 1 s) had to be used with the available maximum tube currents to reach radiation dose levels of 10 and 20 mGy; these doses could not be reached while rotating at 0.28 s. Including the four iodine concentrations (3, 4, 5, and 6%), a total of 3 × 2 × 4 = 24 raw datasets were obtained. Images were then reconstructed using the partly model-based adaptive statistical iterative reconstruction-V (ASiR-V) algorithm with three increasing ASiR-V levels: 0, 50, and 90%. As a result, 72 stacks of CTA images with a spatial resolution of 0.39 × 0.39 × 0.63 mm^3^ were processed and analyzed. In addition, to investigate the effect of voxel size on CSA measurements, images were further reconstructed at two lower spatial resolutions, and analyzed using the same method as for the original high-resolution images. The first set of lower-resolution images was reconstructed with the thickest section available on the scanner (5 mm) while maintaining the same in-plane spatial resolution, thus yielding voxels of 0.39 × 0.39 × 5.0 mm^3^. For the second set, the reconstructed voxel size was further downsampled to 0.63 × 0.63 × 5.0 mm^3^, which approximated the voxel size of MR images.

**Table 1:** Computed tomography angiography (CTA) data acquisition and image reconstruction parameters

CTA Protocol	
Scanner	Revolution CT (GE Healthcare)
Scan mode	Axial
Tube potential	100 and 120 kVp
Tube current (fixed)	320, 360, 500, and 570 mA
Gantry revolution time	0.28, 0.5, and 1 s
Volume CT dose index	5, 10, and 20 mGy
Beam collimation	256 × 0.625 mm
Scan field of view	32 × 32 cm^2^
Display field of view	20 × 20 cm^2^
Section thickness	0.625 mm
Voxel size	0.39 × 0.39 × 0.625 mm^3^
Kernel	Standard
Algorithm	0, 50, and 90% ASiR-V

ASiR-V indicates adaptive statistical iterative reconstruction-V

For the subsequent moving phantom experiment, we only used the minimum gantry revolution time (0.28 s) to properly freeze the phantom motion, together with the following optimized acquisition and reconstruction parameters: 100 kVp and 90% ASiR-V. A second dataset was still acquired at 20 mGy with the same optimized parameters (except for 1-s revolution time), yet these latter images were only used for visual assessment and comparison but not for quantitative analysis.

### CMR protocol

CMR scans were performed on a clinical 3 T system (Magnetom Prisma, Siemens Healthineers, Erlangen, Germany) with 18-channel-chest and 32-channel-spine coil arrays for signal reception. A high-resolution localizer image was first obtained to position the imaging slice orientation perpendicular to the holes of the resolution phantom. CMR images were acquired under both static and moving conditions using a conventional 2D radial, retrospectively-gated, spoiled gradient-recalled echo (SPGRE) cine sequence with the parameters detailed in Table 2 [11, 13]. Although the mock coronary resolution phantom did not simulate epicardial fat, a fast water-selective excitation pulse was nevertheless used so as to keep the protocol consistent with previously reported in vivo studies [11]. The acquisition was repeated 10 times for each of the 8 investigated gadolinium concentrations.

**Table 2:** Cardiovascular magnetic resonance (CMR) acquisition and reconstruction parameters

CMR Protocol	
Scanner	Magnetom Prisma 3 T (Siemens Healthineers)
Sequence	2D radial retrospectively-gated SPGRE
Fat suppression	Water selective excitation pulse
RF excitation angle	22°
TR/TE	4.9/2.7 ms
Pixel bandwidth	570 Hz/pixel
Acquisition time	19.5 s
Temporal resolution	40 ms
Radial views	247 per cardiac phase
Views per segment	19
R-R intervals	13
Field of view	260 × 260 mm^2^
Image matrix	416 × 416 pixels
Pixel size	0.625 × 0.625 mm^2^
Slice thickness	6.5 mm
Radial undersampling	38%

SPGRE, spoiled gradient-recalled echo; RF, radiofrequency; TR/TE, repetition time/echo time

### Image analysis

#### Cross-sectional area measurements

For both CTA and CMR, the CSAs of drilled holes were computed using a previously described [11], fully-automated custom-written software package developed in Matlab (The MathWorks, Natick, Massachusetts, USA). The algorithm automatically detected and segmented the lumen of the simulated coronary arteries using the full-width at half-maximum (FWHM) criterion. For high-resolution CTA images (0.39 × 0.39 × 0.63 mm^3^), 20 sections per dataset were used to compute the CSAs, which yielded 100 CSA measurements per hole diameter, iodine concentration, dose level, tube potential, and reconstruction algorithm; whereas, for lower-resolution CTA images (5-mm thickness), only three sections per dataset could be used to compute the CSAs, yielding only 15 CSA measurements per hole diameter and each investigated CTA parameter. For CMR, two cine frames per dataset were used to compute the CSAs. As a result, a total of 20 frames per investigated gadolinium concentration were analyzed, yielding 100 CSA measurements per hole diameter and gadolinium concentration for analysis.

#### Analysis of cross-sectional area measurements

The measured CSAs were then analyzed using the same method as previously described [13]. The analysis determined both the accuracy and precision of CSA measurements, as well as the limit of detection (LOD) of CSA differences for each imaging modality. The accuracy of CSA measurements was defined as the bias or deviation of the measured CSA from the known drilled CSA, whereas the precision was determined by the standard deviation of CSA measurements for a given diameter [13]. The LOD, which quantifies the sensitivity of the imaging modality to detect small CSA differences, was calculated with a statistical test based on the area under the receiver operating characteristic (ROC) curve (AUC) [13]. Two different hole sizes were considered distinguishable from one another when the AUC was ≥0.95 (no significant overlap of CSA measurement distributions). The LOD was then reported as the smallest CSA difference that could be detected, expressed in both absolute CSA differences (in mm^2^) and differences relative to a 3-mm nominal diameter (in %). In the case where the largest simulated CSA difference (between the smallest and largest investigated hole sizes) could not be detected (AUC <  0.95), the LOD was not reported.

In addition, the SNR and circularity were computed for each segmented hole and every combination of investigated CTA and CMR parameters. For CTA images, noise was defined as the standard deviation (SD) of the CT numbers in regions of interest (ROIs) in the lung equivalent material of the anthropomorphic thorax phantom; whereas for CMR, ROIs were placed in a region void of any signal source. Similarly, the circularity or isoperimetric quotient [22] was computed for each segmented hole. Circularity was defined as *circ* = 4*πA*/*L*^2^, where *circ* ≤ 1, and *A* and *L* are the CSA and perimeter of the segmented hole, respectively.

### Statistical analysis

Data were analyzed with Matlab and GraphPad Prism (GraphPad Software, La Jolla, California, USA). CSA measurements were tested for normal distribution using the Shapiro-Wilk [23] test. Linear regression analyses were used to evaluate the correlation and agreement between the measured and known drilled CSAs. Graphs representing the accuracy, precision, LOD, SNR, and circularity were presented as line plots with error bars showing the mean and SD. For CTA, four-way analysis of variance (ANOVA) was used to test whether the iodine concentration, dose level, tube potential, and reconstruction algorithm had a significant effect on the accuracy, precision, LOD, SNR, and circularity. The ANOVA model computed *p*-values for null hypotheses on the main effects and two-factor interactions, with *post-hoc* Tukey’s tests where appropriate. Similarly, for CMR data, one-way ANOVA with *post-hoc* comparisons were used to assess the effect of gadolinium concentration on the accuracy, precision, LOD, SNR, and circularity. The optimal set of CTA acquisition and reconstruction parameters for the assessment of CEF was determined by selecting the parameters that yielded the smallest LOD, i.e. the highest sensitivity in detecting small CSA differences. The performance of the optimized CTA protocol was then compared with the performance of radial CMR using the independent-samples two-tailed Student’s t-test. Finally, the effect of phantom motion on the accuracy, precision, LOD, SNR, and circularity achieved with CTA and CMR were evaluated using two-way ANOVA and a *post-hoc* Tukey test for multiple comparisons.

## Results

87.9% of CSA measurements with CTA and 76.1% of CMR measurements were well-modeled by a normal distribution

### Optimization of CTA parameters

The iodine concentration, dose level, tube potential, and reconstruction algorithm all had statistically significant effects on the accuracy, precision, LOD, SNR, and circularity (all *p* <  0.001; Table 3). All *post-hoc* comparisons were also statistically significant for the accuracy, precision, LOD, SNR, and circularity (all *p* <  0.001). Furthermore, all two-factor interactions also had significant effects (all *p* ≤ 0.008).

**Table 3:** Analysis of variance (ANOVA) results of the main effects

Accuracy					
Source	Sum of Squares	d.f.	Mean Square	F	*p*
Iodine Concentration	20.4	3	6.8	675.9	< 0.001
Radiation Dose Level	40.3	2	20.2	2003.1	< 0.001
Tube Potential	15.9	1	15.9	1584.1	< 0.001
Reconstruction Algorithm	65.5	2	32.8	3254.6	< 0.001
Precision					
Source	Sum of Squares	d.f.	Mean Square	F	*p*
Iodine Concentration	27.5	3	9.2	3754.9	< 0.001
Radiation Dose Level	14.9	2	7.4	3047.7	< 0.001
Tube Potential	7.7	1	7.7	3153.0	< 0.001
Reconstruction Algorithm	2.1	2	1.0	426.3	< 0.001
LOD					
Source	Sum of Squares	d.f.	Mean Square	F	*p*
Iodine Concentration	68.6	3	22.9	1556.9	< 0.001
Radiation Dose Level	49.9	2	25.0	1700.0	< 0.001
Tube Potential	23.0	1	23.0	1565.7	< 0.001
Reconstruction Algorithm	7.5	2	3.7	254.3	< 0.001
SNR					
Source	Sum of Squares	d.f.	Mean Square	F	*p*
Iodine Concentration	98,595	3	32,865	76,477.7	< 0.001
Radiation Dose Level	159,722	2	79,861	185,839.2	< 0.001
Tube Potential	21,144	1	21,144	49,202.4	< 0.001
Reconstruction Algorithm	64,641	2	32,320	75,210.3	< 0.001
Circularity					
Source	Sum of Squares	d.f.	Mean Square	F	*p*
Iodine Concentration	0.127	3	0.042	1998.5	< 0.001
Radiation Dose Level	0.079	2	0.039	1866.1	< 0.001
Tube Potential	0.032	1	0.032	1520.1	< 0.001
Reconstruction Algorithm	0.021	2	0.010	498.2	< 0.001

Dependent variables: accuracy, precision, limit of detection (LOD), signal-to-noise ratio (SNR), and circularity. Independent variables: iodine concentration, radiation dose level, tube potential, and image reconstruction algorithm. d.f. indicates degrees of freedom

**Fig. 2:**
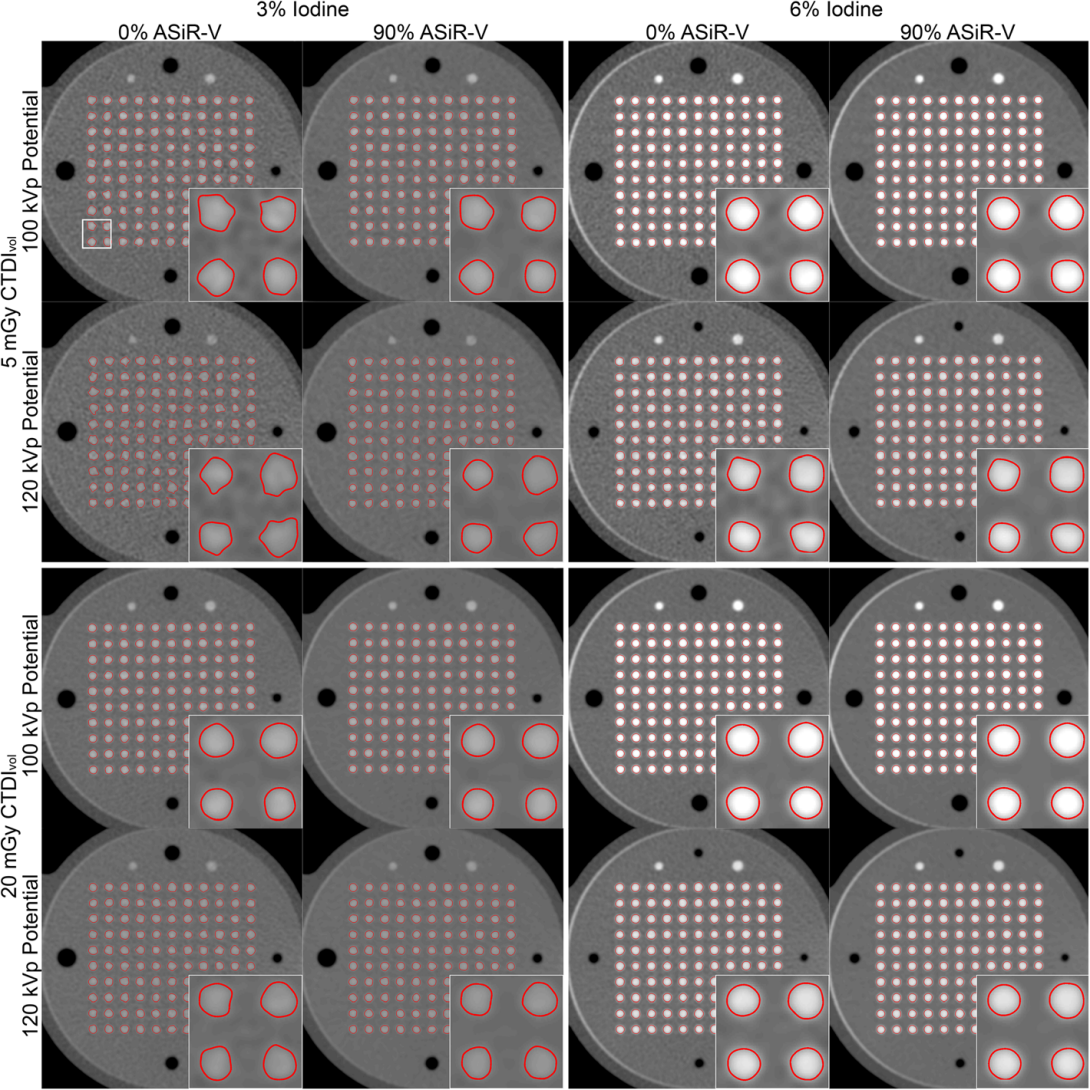
Representative computed tomography angiography (CTA) images of the resolution phantom for a subset of investigated data acquisition and image reconstruction parameters. The segmented cross-sectional areas (CSAs) automatically obtained with the full-width at half-maximum (FWHM) approach are overlaid in red. Images are shown using fixed window width and center of 1000 HU and 200 HU, respectively. ASiR-V indicates adaptive statistical iterative reconstruction-V; CTDI_vol_, volume CT dose index

**Fig. 3:**
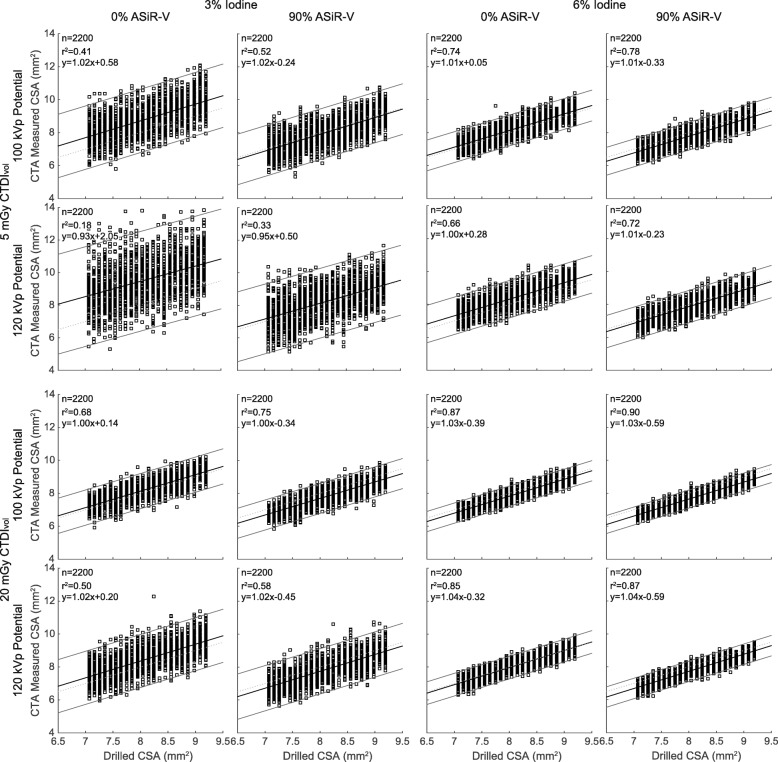
Linear regression analyses between cross-sectional area (CSA) measurements from computed tomography angiography (CTA) images and known drilled CSAs. For readability, only the regressions corresponding to the same subset of CTA data acquisition and image reconstruction parameters as in Fig. 2 are shown. ASiR-V indicates adaptive statistical iterative reconstruction-V; CTDI_vol_, volume CT dose index

**Fig. 4:**
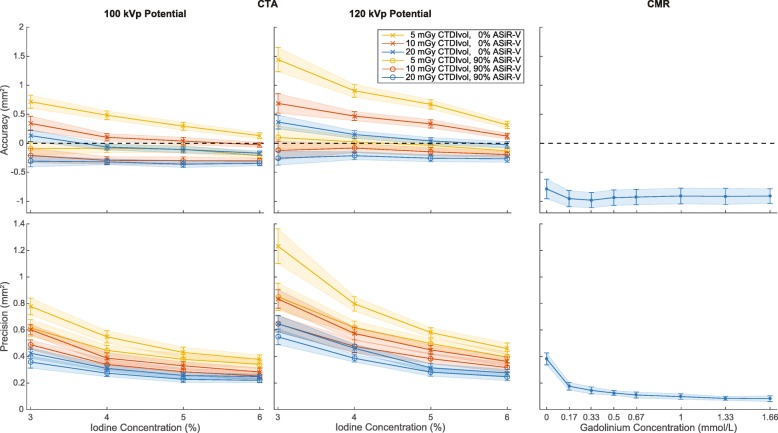
Accuracy and precision of cross-sectional area (CSA) measurements obtained with computed tomography angiography (CTA) and cardiovascular magnetic resonance (CMR) for the different concentrations of contrast media investigated in our study. CTA plots only show the results corresponding to a subset of investigated data acquisition and image reconstruction parameters. Line plots illustrate the mean and standard deviation. ASiR-V indicates adaptive statistical iterative reconstruction-V; CTDI_vol_, volume CT dose index

**Fig. 5:**
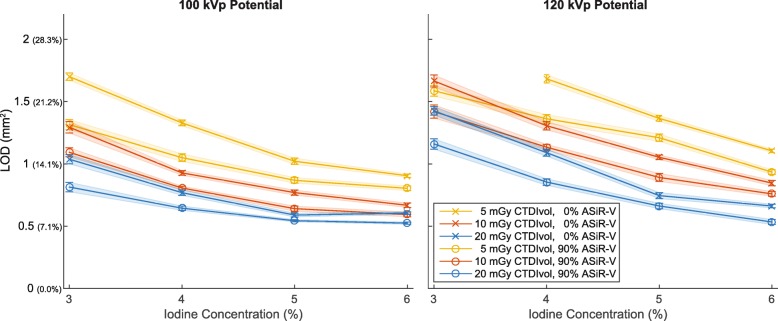
Limit of detection (LOD) or smallest detectable cross-sectional area (CSA) difference obtained with computed tomography angiography (CTA) for a subset of data acquisition and image reconstruction parameters. Note that the point corresponding to 120 kVp, 5 mGy, 0% ASiR-V, and 3% iodine is missing because the largest simulated CSA difference (between the smallest and largest investigated hole sizes) could not be detected (AUC <  0.95) with these parameters. Line plots show the mean and standard error instead of the mean and standard deviation for readability of the plots. For the reader’s convenience, the LOD axis (vertical axis) is provided in absolute CSA differences (in mm^2^) and difference relative to a 3-mm nominal diameter (in %) in parenthesis. ASiR-V indicates adaptive statistical iterative reconstruction-V; CTDI_vol_, volume CT dose index

All CTA parameters had a visual effect on the quality of CSA segmentations (Fig. 2). There was a significant correlation between measured and drilled CSAs for each combination of investigated data acquisition and image reconstruction parameters (all *p* <  0.001). The agreement (r^2^) ranged from 0.18 to 0.90 and improved substantially with higher iodine concentration, higher dose level, lower tube potential, and higher percentage of ASiR-V (Fig. 3). On the other hand, the slopes of the regression analyses remained very close to unity for any combination of CTA parameters, ranging from 0.93 to 1.04. The accuracy, or bias, of measured CSAs significantly depended on all parameters (all *p* <  0.001). We also found that higher iodine concentration, higher dose level, lower tube potential, and higher percentage of ASiR-V yielded smaller absolute CSA measurements (Figs. 3 and 4). The precision of CSA measurements significantly increased with higher iodine concentration, higher dose level, lower tube potential, and higher percentage of ASiR-V (all *p* <  0.001). The highest precision, or lowest spread, was achieved with 6% iodine, 20 mGy, 100 kVp, and 90% ASiR-V (Fig. 4). The LOD also significantly depended on the iodine concentration, dose level, tube potential, and reconstruction algorithm (all *p* <  0.001). Similar to the precision curves, the LOD curves in Fig. 5 showed significant improvements with higher iodine concentration, higher tube voltage, lower tube potential, and higher percentage of ASiR-V (all *p* <  0.001). The smallest LOD, or highest sensitivity in detecting small CSA changes, was also achieved with 6% iodine, 20 mGy, 100 kVp, and 90% ASiR-V. This set of acquisition and reconstruction parameters was therefore deemed optimal for the assessment of CEF with CTA, and was subsequently used to compare its performance with CMR.

**Fig. 6:**
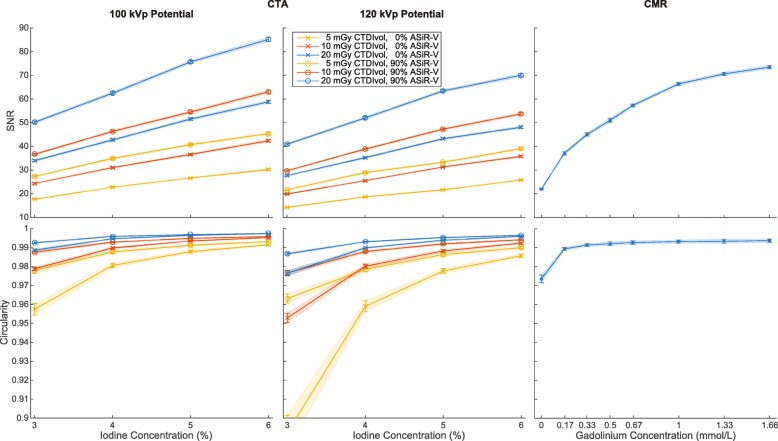
Signal-to-noise ratio (SNR) and circularity of cross-sectional area (CSA) measurements obtained with computed tomography angiography (CTA) and cardiovascular magnetic resonance (CMR) for the different concentrations of contrast media investigated in our study. CTA plots only show the results corresponding to a subset of investigated data acquisition and image reconstruction parameters. Line plots illustrate the mean and standard deviation. ASiR-V indicates adaptive statistical iterative reconstruction-V; CTDI_vol_, volume CT dose index

Similar to precision, SNR and circularity significantly improved with higher iodine concentration, higher dose level, lower tube potential, and higher percentage of ASiR-V (all *p* <  0.001; Fig. 6).

**Fig. 7:**
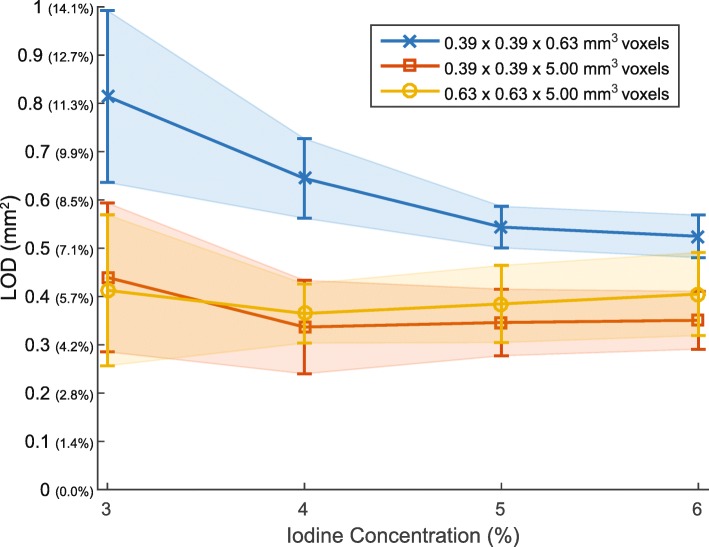
Limit of detection (LOD) or smallest detectable cross-sectional area (CSA) difference obtained using the optimized computed tomography angiography (CTA) protocol for the different voxel sizes investigated in our study. Line plots show the mean and standard deviation. For the reader’s convenience, the LOD axis (vertical axis) is provided in absolute CSA differences (in mm^2^) and difference relative to a 3-mm nominal diameter (in %) in parenthesis

The voxel size had an effect on the LOD for the optimal set of CTA parameters as determined above (Fig. 7). Using 5-mm-thick sections while preserving the high in-plane spatial resolution significantly improved the LOD at every investigated iodine concentration compared with the nominal 0.625-mm section thickness (*p* <  0.001). However, further reducing the in-plane resolution to 0.63 × 0.63 × 5.0 mm^3^ to approximate the CMR voxel size did not significantly change the LOD (*p* = 0.79).

### Comparison of CTA and CMR for CEF assessment

**Fig. 8:**
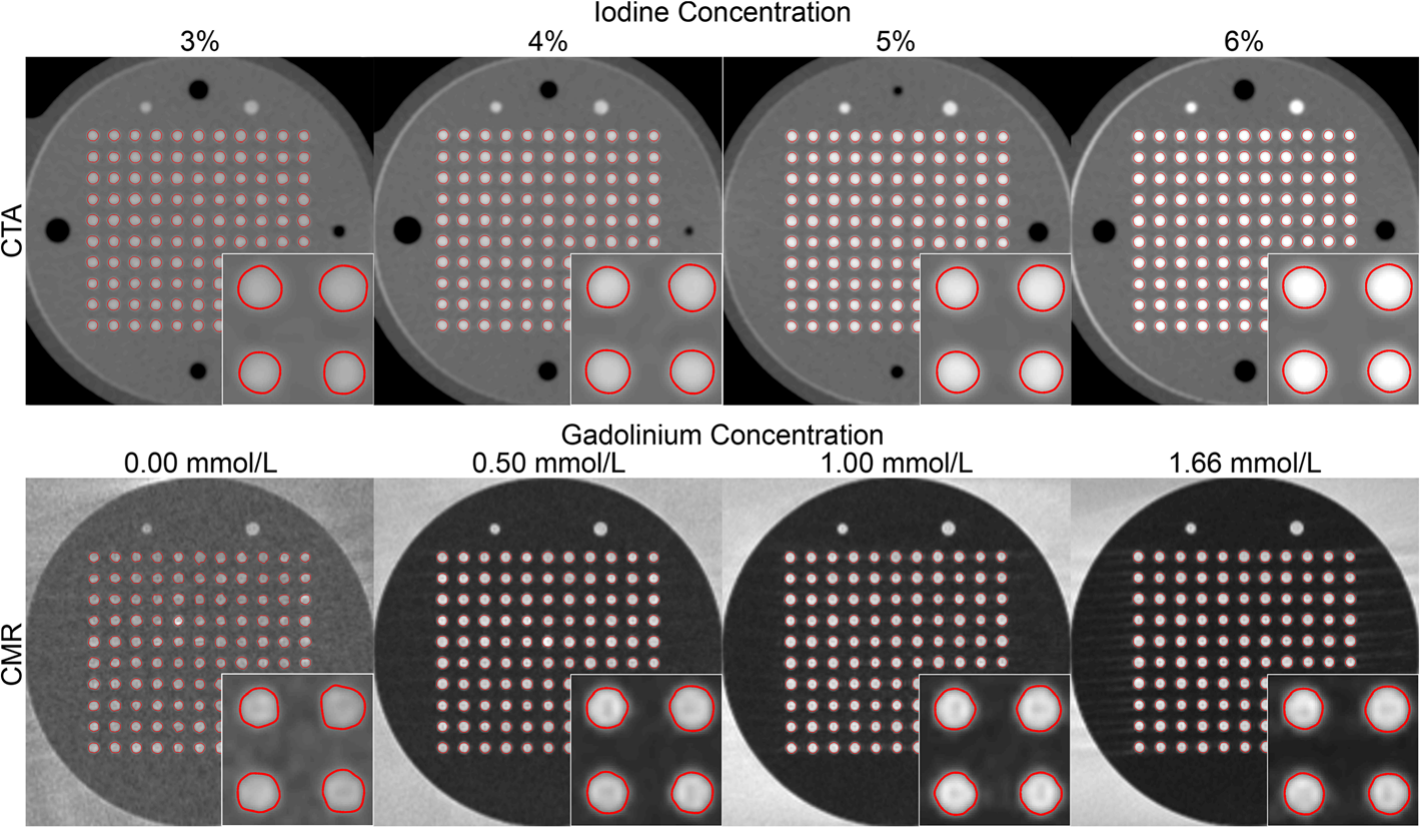
Comparison of representative segmented computed tomography angiography (CTA) and cardiovascular magnetic resonance (CMR) images obtained using the optimized CTA protocol, i.e. 20 mGy, 100 kVp, and 90% ASiR-V. For clarity, only four of the eight gadolinium concentrations are shown for MR images, i.e., 0.00, 0.50, 1.00, and 1.66 mmol/L

**Fig. 9:**
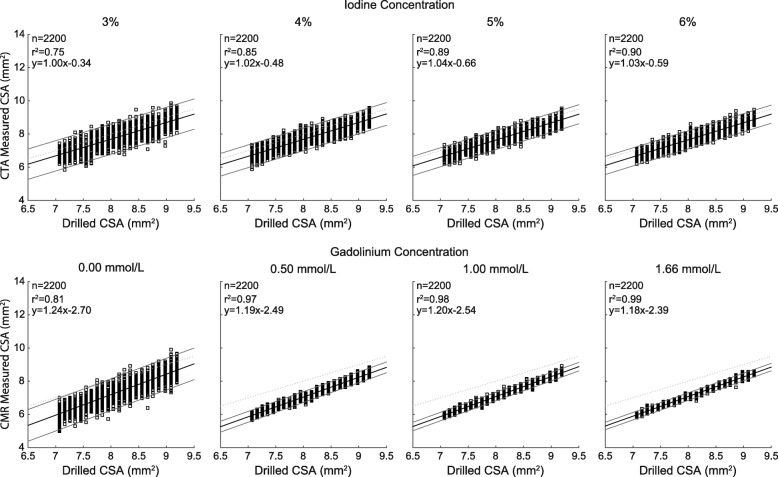
Linear regression analyses of cross-sectional area (CSA) measurements obtained using the optimized computed tomography angiography (CTA) protocol and cardiovascular magnetic resonance (CMR). For readability, only the regressions corresponding to the same subset of images as in Fig. 8 are shown

Representative CMR images were compared with thin-section CTA images obtained using the above determined optimal CTA protocol (Fig. 8). Linear regressions showed a significant correlation between measured and known drilled CSAs for both CTA and CMR (all *p* <  0.001; Fig. 9). The slopes of regression analyses ranged from 1.00 to 1.04 and 1.18 to 1.24 with r^2^ ≥ 0.75 and 0.81 for CTA and CMR, respectively.

CTA measurements were more accurate (less biased) than CMR measurements, regardless of contrast medium concentration (Fig. 4). The measured CSAs with CMR always underestimated the known CSAs and were always smaller than CTA measurements for each investigated concentration. The precision of CTA and CMR measurements both significantly improved with higher contrast medium concentrations (Fig. 4). However, for the optimized CTA protocol, there were no significant differences between 5 and 6% iodine concentrations (*p* = 0.79). There were also no significant differences between gadolinium concentrations higher than 0.50 mmol/L (*p* ≥ 0.50). Finally, for gadolinium concentrations higher than 0.17 mmol/L, CMR measurements were always more precise than CTA measurements, regardless of iodine concentration.

**Fig. 10:**
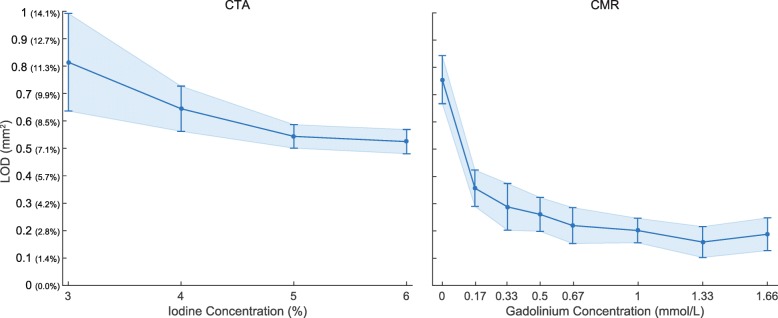
Limit of detection (LOD) or smallest detectable cross-sectional area (CSA) difference obtained using the optimized computed tomography angiography (CTA) protocol and cardiovascular magnetic resonance (CMR) for the different concentrations of contrast media investigated in our study. Line plots show the mean and standard deviation. For the reader’s convenience, the LOD axis (vertical axis) is provided in absolute CSA differences (in mm^2^) and difference relative to a 3-mm nominal diameter (in %) in parenthesis

Similar to precision results, for gadolinium concentrations higher than 0.17 mmol/L, CMR measurements always yielded significantly superior LOD than CTA measurements, regardless of iodine concentration (*p* <  0.001; Fig. 10). The best LOD with CMR was achieved for a gadolinium concentration of 1.33 mmol/L and indicates that, at this concentration, CMR is capable of distinguishing CSA differences of 0.16 ± 0.06 mm^2^, which corresponds to CSA percentage changes of 2.3 ± 0.8% for a 3-mm reference hole diameter. However, the differences between the four highest concentrations (≥ 0.67 mmol/L) were not significant (*p* ≥ 0.06). In comparison, CTA was only capable of distinguishing CSA differences of 0.52 ± 0.04 mm^2^ (7.4 ± 0.6%) at the highest investigated iodine concentration of 6%. However, there were no significant differences between 5 and 6% concentrations (LOD 5% = 0.54 ± 0.04 mm^2^; LOD 6% = 0.52 ± 0.04 mm^2^; *p* = 0.15).

The SNR and circularity values for CTA and CMR measurements strongly depended on the contrast medium concentration (all *p* <  0.001) and were maximum for the highest concentrations (Fig. 6). The maximum SNR with CTA was significantly higher than with CMR (CTA = 85.2 ± 0.8; CMR = 73.5 ± 0.7; *p* <  0.001). Similarly, the maximum circularity was also significantly higher for CTA than CMR (CTA = 0.9974 ± 0.0002; CMR = 0.9937 ± 0.0009; *p* <  0.001).

### Comparison of static and moving CSA measurements

**Fig. 11:**
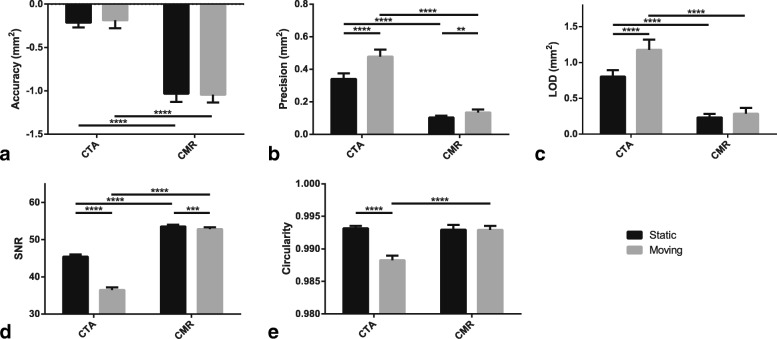
Box-and-whisker plots comparing static and moving phantom experiments. **a** Accuracy, **b** precision, **c** limit of detection (LOD), **d** signal-to-noise ratio (SNR), and **e** circularity of cross-sectional area (CSA) measurements for computed tomography angiography (CTA) and cardiovascular magnetic resonance (CMR). For this comparison, CTA data were acquired using 5 mGy, 100 kVp, and 6% iodine, and reconstructed with 90% adaptive statistical iterative reconstruction-V (ASiR-V). ** indicates *p* ≤ 0.01; ***, *p* ≤ 0.001; ****, *p* ≤ 0.001

**Fig. 12:**
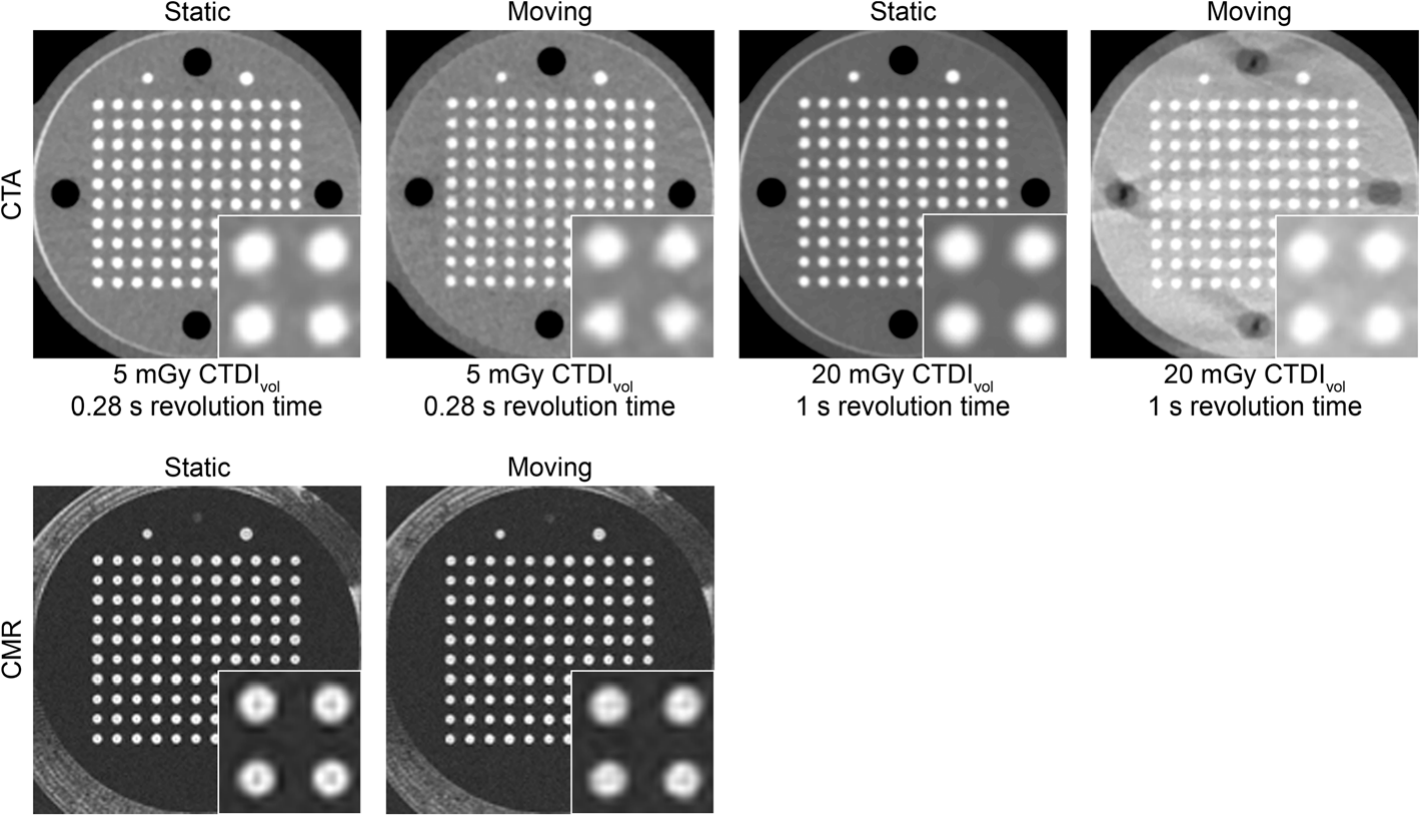
Comparison of representative computed tomography angiography (CTA) and cardiovascular magnetic resonance (CMR) images obtained under static and moving conditions. CTA data were acquired using 5 or 20 mGy (with adaptation of the gantry revolution time), 100 kVp, and 6% iodine, and reconstructed with 90% ASiR-V. CTDI_vol_ indicates volume CT dose index

The accuracy of CSA measurements did not significantly differ between static and moving experiments for both CTA (*p* = 0.22) and CMR (*p* = 0.67) (Fig. 11a). However, CTA and CMR measurements were both significantly more precise under static conditions (*p* <  0.001; Fig. 11b). Similarly, CTA and CMR measurements yielded superior LOD for the static relative to the moving experiment, though without reaching statistical significance for CMR (*p* <  0.001 and *p* = 0.06, respectively; Fig. 11c). Measurements under moving conditions also yielded significantly lower SNR for both CTA and CMR (*p* <  0.001; Fig. 11d). Finally, only CTA circularity measurements were significantly decreased during the moving experiment (*p* <  0.001; Fig. 11e). Differences in precision (0.14 mm^2^ vs. 0.03 mm^2^), LOD (0.37 mm^2^ vs. 0.05 mm^2^), SNR (8.9 vs. 0.7), and circularity (0.0049 vs. 0.0002) between static and moving measurements were all larger with CTA than CMR. Figure 12 illustrates the effect of the gantry revolution time in presence of motion, and clearly shows that the slowest revolution time (1 s) failed to properly freeze motion during CTA data acquisition. Visually, the moving CTA images for the fastest revolution time (0.28 s) also exhibited more motion artifacts than the moving MR images, even though their overall acquisition time was substantially shorter (a single heartbeat vs. multiple cardiac cycles, respectively).

## Discussion

### Optimization of CTA parameters

Our results first showed that the investigated CTA data acquisition and image reconstruction parameters all had significant effects on the measured quantities. Among the CTA parameters investigated in this in vitro 256-multidetector CT study, the best settings for the assessment of CEF were 20 mGy, 100 kVp, and 90% ASiR-V. With this optimized protocol, coronary artery CSA differences of 0.52 ± 0.04 mm^2^ (7.4 ± 0.6%) could be reliably identified under static conditions. However, according to the trends visible in Fig. 5, it is expected that higher iodine concentration, higher dose level, lower tube potential, and higher percentage of ASiR-V may further slightly improve the LOD of CTA.

With 6% iodine concentration, the x-ray attenuation difference between the contrast medium blend and PMMA resolution phantom approximately simulated the contrast measured in vivo between the blood from the coronary artery lumen and the surrounding pericardial fat. Based on the trend of the LOD curve for the optimized CTA protocol in Fig. 10, it can be expected that higher iodine concentrations would further slightly improve the LOD of CTA; however, because the curve appears to reach a plateau, it is unclear if this would lead to any significant improvement. Indeed, increasing the concentration from 5 to 6% improved the LOD, but the difference was not significant (*p* = 0.15). In addition, the potential risk of contrast-induced nephropathy is driving CT examinations toward a restricted use of contrast media to the smallest diagnostically appropriate amount [24, 25].

Higher radiation doses could further improve the LOD of CTA (Fig. 5). However, to assess the CEF in vivo, CTA scans must be repeated at rest and under stress to measure the flow-mediated CSA changes of coronary arteries and, thus, double the overall examination dose. For this reason, the use of higher doses would exceed the most recent diagnostic reference levels for coronary CTA [26–28] and is, thus, not clinically justifiable. In addition, for cardiac imaging applications, one should select the lowest acquisition time available to minimize motion blurring. Using a gantry revolution time of 0.28 s with a data acquisition angle of 180° would result in a 0.14 s acquisition time for the single heartbeat case. Knowing that the normalized CTDI_vol_ is in the range of 0.1 mGy per mAs, using a CTDI_vol_ of 20 mGy would require a tube current over 1.4 A, which is currently not available on clinical CT systems.

A further trend visible in Fig. 5 is the improvement of the LOD with higher percentages of ASiR-V reconstruction. It is possible for a fully model-based iterative reconstruction algorithm, such as Veo (General Electric Healthcare) [29], to outperform the results presented here. When compared with three other algorithms from a single CT manufacturer, including ASiR-V, recent studies demonstrated that Veo resulted in superior spatial resolution and detectability [30, 31]. Unfortunately, Veo is currently not available on our 256-multidetector CT system and could, therefore, not be compared in this study.

CTA offers higher 3D spatial resolution than CMR and, therefore, can potentially resolve finer details. However, the use of smaller voxels leads to fewer photons per voxel and thus results in images with decreased SNR, which may mitigate the effect of the improved spatial resolution. As a result, we also investigated the effect of voxel size on the measured CSAs. Our results indicated that the LOD significantly improved with thicker sections (Fig. 7). These results seem to suggest that, for in vivo coronary CTA and the specific clinical task of CEF assessment, higher SNR should be preferred over higher spatial resolution to enable more precise measurements of coronary artery morphology and the detection of smaller CSA differences. However, several issues arise from generating thicker images with CT. Unlike CMR, which can acquire images in any orientation, CT is restricted to acquire images in the axial (or slightly axial-oblique) plane. Since coronary arteries are unlikely to be perpendicular to the acquired section over their entire length, multiplanar reformatted images must be reconstructed by interpolating the nonisotropic CT dataset to measure the coronary CSA. Additionally, coronary arteries have most often complex geometries and, therefore, CSA measurements from thick-section multiplanar reformations can be affected by the artery obliquity and tortuosity.

### Comparison of CTA and CMR for CEF assessment

Our second objective was to compare the performance of radial coronary CMR with the optimized CTA protocol for the assessment of CEF, which mainly requires a higher precision (rather than accuracy) of CSA measurements. We found that CMR was capable of detecting significantly smaller CSA differences than CTA for gadolinium concentrations higher than 0.17 mmol/L. Radial CMR could distinguish CSA differences in the order of 0.16 ± 0.06 mm^2^ for images with high SNR (~ 70), which corresponds to a coronary CSA percentage difference of 2.3 ± 0.8% for a 3-mm baseline diameter. For low SNR (~ 22) images, radial CMR was able to reliably detect CSA differences of 0.75 ± 0.09 mm^2^ (10.7 ± 1.3%). These results are in good agreement with earlier work [13] and suggest that radial CMR with sufficiently high SNR is suitable for measuring CSA differences in the range of previously reported endothelium-dependent vasomotor response of proximal coronary arteries in healthy adult subjects (10–25%) [2, 5, 6, 8–10]. In contrast, CTA could only reliably distinguish CSA differences of 0.52 ± 0.04 mm^2^ (7.4 ± 0.6%) for the highest SNR (~ 85) at 6% concentration. At the lowest investigated concentration (SNR ~ 50), the LOD was 0.81 ± 0.04 mm^2^ (11.5 ± 0.5%). The CSA difference detectability of CTA was very limited and arguably insufficient to properly differentiate normal from abnormal endothelial responses. While well supported by the results presented in this study, the poor performance of CTA compared with CMR for this specific clinical task is counter-intuitive and not consistent with our initial hypothesis. From the data presented here, it is unclear why CMR significantly outperformed CTA and further investigations are necessary to address this question in greater detail. This result is even more surprising considering the fact that CTA measurements yielded higher circularity and SNR than CMR measurements. It remains to be seen whether these results can be explained, at least in part, by partial volume effect and/or changes in noise texture [32]. Although these results indicate that CMR theoretically enables the detection of smaller CSA differences than CTA, they must be interpreted with caution since the much larger (ten-fold) slice thickness associated with the 2D CMR protocol may decrease the precision of CSA measurements when the coronary artery segment is not perfectly perpendicular to the prescribed slice unlike in this in vitro phantom study. However, the area measurement is inversely proportional to the cosine of the angle between the misaligned plane and the perpendicular cross section and, therefore, does not change significantly for small misalignments. For instance, a misalignment of 14 degrees would lead to a ~ 3% error in area, which approximates the smallest CSA differences detectable with CMR.

CSA was systematically underestimated by CMR. This observation is consistent with previous studies [13, 21] that also used a similar FWHM algorithm for segmentation. As demonstrated by Schar et al. [21], CSA measurements were underestimated in both numerical simulations and phantom studies, and the underestimation was worse with image interpolation. Hoogeveen et al. [33] also showed that the FWHM algorithm may underestimate area measurements. Although strictly the same segmentation algorithm was used to segment both CTA and CMR images, CSA was not consistently underestimated by CTA.

### Comparison of static and moving CSA measurements

The results of the moving phantom experiments indicate that CMR was less affected by motion than CTA in terms of precision, LOD, SNR, and circularity of CSA measurements. In other words, except for accuracy, the differences between static and moving quantitative parameter values were smaller for CMR than CTA. It should be noted that, while the overall acquisition time with CTA was much shorter than with CMR (a single vs. 13 cardiac cycles), the effective acquisition window was shorter with CMR. The duration of the acquisition window determines the amount of displacement and, thus, the amount of motion blurring. The displacement experienced by the phantom during the acquisition window can be analytically quantified for both CTA and CMR. Specifically, the acquisition window for CTA corresponds to half of the gantry revolution time (${\mathrm{T}}_{{\mathrm{window}}_{\mathrm{CT}}}=\frac{280\ \mathrm{ms}}{2}=140\ \mathrm{ms}$), while the acquisition window for CMR is determined by the number of views per segment *N*_*views*_ = 19 and the echo spacing TR = 5 ms (${\mathrm{T}}_{{\mathrm{window}}_{\mathrm{MR}}}={N}_{views}\mathrm{TR}=95\ \mathrm{ms}$). Given the time-dependent position of the moving phantom simulating a unidirectional sinusoidal cardiac rhythm *x*(*t*) = *A* sin(*ωt*), where *A* = 1.5 cm, *ω* = 2*π*/*T*, and *T* = 1.0 s (60 beats per minute), one can compute the displacement occurring during the acquisition window, which will lead to blurring in the reconstructed images. To minimize motion artifacts, the acquisition window should be centered with the extreme position of the moving phantom where it moves the least (*t*^′^ = *T*/4). While this represents the most favorable situation, it illustrates the potential displacement experienced by the moving phantom during the acquisition window. Under such conditions, the displacement is given by *∆*_*x*_ = |*x*(*t*^′^ ± *T*_*window*_/2) − *x*(*t*^′^)| = *A*|sin(*ω*(*t*^′^ ± *T*_*window*_/2)) − sin(*ωt*^′^)|, which is equal to 1.4 mm and 0.6 mm for CTA and CMR acquisitions, respectively. In other words, the amount of displacement during the CTA acquisition window was more than twice the displacement occurring during its CMR counterpart. This observation at least partly explains why CMR outperformed CTA in the presence of motion. It should also be noted that MR images were collected using segmented techniques acquired over multiple cardiac cycles. The beat-to-beat repositioning precision of the coronary artery has previously been shown to be < 1 mm [34], which implies that the repositioning misalignment could further negatively affect the performance of CMR.

### Limitations

The CMR experiments presented in this study are similar to the ones reported in an earlier study [13] and were repeated here to add a rigorous comparison with CTA. CTA and its comparison to CMR was not part of this earlier study. In the present report, the shape and material of the resolution phantom were adapted. Specifically, the phantom was shaped in the form of a cylinder to be inserted into a commercially available anthropomorphic thorax phantom (QRM) and to minimize x-ray edge-enhancement artifacts. In addition, the phantom was made of PMMA instead of polyoxymethylene copolymer to more closely simulate fat attenuation. Finally, this study used different gadolinium concentrations to simulate various SNRs instead of generating artificial noise.

While the limitations of using a phantom to analyze the sensitivity of radial CMR to measure small CSA differences of coronary arteries have already been discussed extensively in [13], there are further limitations pertaining to CTA that are worth mentioning. First, it is possible that different multidetector row CT systems could outperform the one investigated here. In fact, a recent study showed that the detectability indexes of different diameter structures can vary significantly (as much as 283%) across different CT systems and manufacturers [35]. A similar comparative study of multiple CT systems with the resolution phantom used in our study would prove very valuable. However, such a comparison is beyond the scope of this initial investigation. Also, dual-source dual-energy CT systems may prove to be a better option for this specific clinical task owing to their faster gantry revolution time. Second, the effect of various reconstruction kernels was also beyond the scope of this study and, therefore, not investigated. The use of edge-enhancing sharper kernels may further improve the performance of CTA, even though they are not yet routinely used in clinical practice for coronary CTA. Recent in vitro studies showed that dedicated sharp kernels for multi-energy photon-counting CT combined with iterative reconstruction techniques yielded superior qualitative and quantitative images of coronary stents compared with conventional CT reconstruction techniques [18, 36]. Third, it is possible that the resolution phantom was not perfectly aligned with the scanner during CTA data acquisition. Such misalignment would introduce a bias in the measured CSAs and could partly explain why CTA yielded larger CSAs than CMR. However, special care (the same as for CMR) was taken to precisely align the phantom using CT localizer radiographs. Fourth, we did not evaluate the performance of dual- and multi-energy CT techniques, which were not available on our system at the time of the study. Our multiparametric quantitative CT analysis may, however, pave the way for the subsequent optimization of dual- and multi-energy coronary CTA, which offers higher SNR and dose-corrected contrast-to-noise ratio with fewer beam hardening artifacts, and potential for iodine concentration reduction with virtual monoenergetic images reconstructed closer to the k-edge of iodine [37–40]. Fifth, because of the very short spin-spin relaxation time of PMMA, the phantom was not visible on CMR images and, therefore, simulated ideal in vivo coronary imaging by assuming perfectly suppressed signal from the pericardial fat surrounding coronary arteries. Although a different material visible on CMR images would have been preferred, there is, to our knowledge, no such material that has both the mechanical and nuclear magnetic resonance properties that enable very accurate drilling while also emitting an CMR signal. This study design thus slightly favors CMR in comparison to CT to some extent due to zero background CMR signal. Finally, it should be noted that the moving phantom experiment only simulated rigid motion, while coronary artery motion over the entire cardiac cycle is highly non-rigid, which could therefore slightly affect the performance results.

## Conclusions

We present a phantom study with well-controlled boundary conditions to compare the performance of 256-multidetector CTA and radial CMR for the in vitro quantification and detection of simulated coronary artery CSA differences. Although unexpected, our results effectively support that radial coronary CMR was more precise and outperforms CTA for the specific diagnostic task of detecting small CSA differences. However, CTA yielded more accurate CSA measurements, which may prove useful in other clinical scenarios. This suggests that radial CMR is suitable for measuring CSA differences an order of magnitude smaller than those reported for healthy physiological vasomotor responses of proximal coronary arteries. Together with isometric handgrip exercise, CMR might thus offer a noninvasive, safe, and quantitative technique to assess CEF and study atherosclerosis progression or response to therapy.

## Abbreviations

ANOVA: Analysis of variance
ASiR-V: Adaptive statistical iterative reconstruction-V
AUC: Area under the ROC curve
CEF: Coronary endothelial function
CMR: Cardiovascular magnetic resonance
CNR: Contrast-to-noise ratio
CSA: Cross-sectional area
CT: Computed tomography
CTA: Computed tomography angiography
CTDI_vol_: Volume CT dose index
FWHM: Full width at half maximum
HU: Hounsfield unit
LOD: Limit of detection
PMMA: Polymethyl methacrylate
RF: Radiofrequency
ROC: Receiver operating characteristic
SD: standard deviation
SNR: Signal-to-noise ratio
SPGRE: Spoiled gradient-recalled echo
TE: Echo time
TR: Repetition time
